# Combined Anticancer Effect of Plasma-Activated Infusion and Salinomycin by Targeting Autophagy and Mitochondrial Morphology

**DOI:** 10.3389/fonc.2021.593127

**Published:** 2021-06-04

**Authors:** Takashi Ando, Manami Suzuki-Karasaki, Miki Suzuki-Karasaki, Jiro Ichikawa, Toyoko Ochiai, Yukihiro Yoshida, Hirotaka Haro, Yoshihiro Suzuki-Karasaki

**Affiliations:** ^1^ Department of Orthopaedic Surgery, Yamanashi University School of Medicine, Yamanashi, Japan; ^2^ Department of Research and Development, Plasma ChemiBio Laboratory, Plasma ChemiBio Laboratory, Nasushiobara, Tochigi, Japan; ^3^ Department of Dermatology, Nihon University Hospital, Tokyo, Japan; ^4^ Department of Orthopaedic Surgery, Nihon University School of Medicine, Nihon University Orthopaedic Surgery, Tokyo, Japan

**Keywords:** cold atmospheric pressure plasma, plasma-activated infusion, tumor-targeted therapy, salinomycin, necroptosis, autophagy, mitochondrial network

## Abstract

Non-thermal atmospheric pressure plasma (NTAPP)-activated liquids have emerged as new promising anticancer agents because they preferentially injure malignant cells. Here, we report plasma-activated infusion (PAI) as a novel NTAPP-based anti-neoplastic agent. PAI was prepared by irradiating helium NTAP to form a clinically approved infusion fluid. PAI dose-dependently killed malignant melanoma and osteosarcoma cell lines while showing much lower cytotoxic effects on dermal and lung fibroblasts. We found that PAI and salinomycin (Sal), an emerging anticancer stem cell agent, mutually operated as adjuvants. The combined administration of PAI and Sal was much more effective than single-agent application in reducing the growth and lung metastasis of osteosarcoma allografts with minimal adverse effects. Mechanistically, PAI explicitly induced necroptosis and increased the phosphorylation of receptor-interacting protein 1/3 rapidly and transiently. PAI also suppressed the ambient autophagic flux by activating the mammalian target of the rapamycin pathway. PAI increased the phosphorylation of Raptor, Rictor, and p70-S6 kinase, along with decreased LC3-I/II expression. In contrast, Sal promoted autophagy. Moreover, Sal exacerbated the mitochondrial network collapse caused by PAI, resulting in aberrant clustering of fragmented mitochondrial in a tumor-specific manner. Our findings suggest that combined administration of PAI and Sal is a promising approach for treating these apoptosis-resistant cancers.

## Introduction

Osteosarcomas (OS) are malignant bone tumors accompanied by tumorous cartilage/bone or osteoid matrix formation. These tumors account for 15–20% of all malignant bone tumors, accounting for the highest percentage of all tumors (1, 2). The incidence of OS is relatively low at 4.8 new cases per 1,000,000 individuals; however, most patients are children/adolescents aged <20 years. According to the histological grade, OS stages are classified as stage I (low-level malignancy) and stage II (high-level malignancy). The standard treatment for stage I tumors is surgery alone. Stage II or higher-grade tumors are therapeutically problematic. Standard treatment for stage IIA, IIB, or III tumors is a multidisciplinary approach consisting of pre-and post-operative chemotherapy and surgery. The 3-year overall survival rate ranges from 61% to 82%. For OS, combination chemotherapy with methotrexate, doxorubicin, and cisplatin is primarily used. However, 35–45% of patients with OS are insensitive to chemotherapy, and their 5-year survival rate is only 5–20% (3). No other new drug with a high response rate has been developed, and it remains challenging to improve prognosis using conventional chemotherapeutic regimens. Thus, an innovative therapy for overcoming drug resistance in OS is urgently required.

Non-thermal atmospheric pressure plasma (NTAPP) injures various tumor cell types while sparing their non-transformed counterparts under optimal conditions (4–7). These properties have attracted much attention in cancer therapy. As with standard direct NTAPP treatment, plasma-activated liquids (PALs) such as NTAPP-irradiated media, solutions, and buffers exhibit vigorous anticancer activity (8–10). Of these, plasma-activated medium (PAM) has been the most widely studied. PAM is conventionally produced by subjecting culture media such as Dulbecco’s modified Eagle’s medium (DMEM) to NTAPP irradiation. Like NTAPP, PAM has a high potential to kill cancer cells, including malignant melanoma (MM) and OS cells, and evidence suggests that it acts in a tumor-specific manner (11–13). However, conventional PAM includes many media components, such as the pH indicator phenol red. This compound undergoes specific metabolism to form a toxic substance in the liver (14). Thus, PAM may damage the liver in patients. As an alternative and safer NTAPP-based tool, we developed a plasma-activated infusion (PAI) by irradiating a clinically approved infusion fluid with NTAPP. As the infusion fluid consists of glucose, lactate, sodium chloride, and potassium chloride, the resulting PAI potentially contains no toxic substances and is expected to be safe for clinical use.

Autophagy is a primary catabolic process in which cellular components and damaged organelles undergo degradation. Three different types of autophagy have been identified: macroautophagy (referred to hereafter as autophagy), microautophagy [autophagy of organelles such as mitochondria and endoplasmic reticulum (ER)], and chaperone-mediated autophagy. Autophagy is a complicated process involving induction of a phagophore in the cytoplasm, elongation and autophagosome formation, the fusion of the autophagosome with lysosomes, and the degradation of autophagosomal contents (15–17). These events are strictly controlled by autophagy-related genes (15). Autophagy copes with cellular stresses such as starvation and supplies energy and metabolic precursors. It is negatively regulated by the mammalian target of the rapamycin complex 1/2 (mTORC1/2) in response to insulin and amino acid signals. During nutrient deprivation, this negative regulation by mTORC1/2 is alleviated, resulting in autophagy induction (15, 18, 19). Autophagy also contributes to removing damaged organelles such as mitochondria and ER *via* mitophagy and ERphagy, respectively. Accordingly, autophagy is essential for cancer cells’ survival by satisfying their high-energy demands and removing damaged organelles (20, 21). However, intensive and persistent autophagy activation leads to programmed cell death, known as autophagic cell death (22–24).

Salinomycin (Sal) is a naturally occurring polyether antibiotic that acts on potassium and calcium ionophores and has been used as an anticoccidial agent in the poultry industry for a long time. Sal has recently been considered a promising anticancer drug because of its capacity to selectively kill cancer stem cells and multidrug-resistant cancer cells (25–27). Several reports have demonstrated the participation of autophagic cell death in Sal’s anticancer effect (28–31). In contrast, inhibition of autophagy and the induction of apoptosis play a critical role in Sal’s anticancer effect (32–35) in other studies.

Different anticancer drugs, including temozolomide, epirubicin, and sorafenib, induce autophagy, contributing to drug resistance in various cancer cell types (36–39). Autophagy also contributes to resistance to tumor necrosis factor-related apoptosis-inducing ligand (TRAIL) in colorectal cancers and hepatoma (40–42). We have previously demonstrated that TRAIL induces robust autophagic flux in OS and MM cells whose suppression increases TRAIL-induced apoptosis (43, 44). Together, autophagy suppression represents a promising approach for overcoming drug resistance in cancer cells. Our preliminary data indicate that PAI can injure TRAIL-resistant OS and MM cells. Moreover, PAI can mimic the biological activities of simultaneous administration of TRAIL and autophagy inhibitors. We predicted that PAI could elicit anticancer activity by compromising autophagy. Additionally, if Sal can modulate autophagy, PAI and Sal may function cooperatively. This study was conducted to test these hypotheses in MM and OS cells.

## Materials and Methods

### Materials

All chemicals were purchased from Sigma Aldrich (St. Louis, MO, USA) unless otherwise specified. Soluble recombinant human TRAIL was obtained from Enzo Life Sciences (Farmingdale, NY, USA). The pan-caspase inhibitor Z-VAD-FMK was purchased from Merck Millipore (Darmstadt, Germany). All insoluble reagents were dissolved in dimethyl sulfoxide and diluted with high glucose-containing DMEM supplemented with 10% fetal bovine serum (FBS) or Hank’s balanced salt solution (pH 7.4, Nissui Pharmaceutical Co., Ltd., Tokyo, Japan) (final dimethyl sulfoxide concentration, <0.1%) before use.

### Cell Culture

The human melanoma cell line A2058 (cell number IFO 50276) was obtained from the Japanese Collection of Research Bioresources (JCRB) Cell Bank of National Institutes of Biomedical Innovation, Health, and Nutrition (Osaka, Japan). Human fetal lung fibroblasts, WI-38 (cell number JCRB9017), were obtained from JCRB. Human dermal fibroblasts from the facial dermis were obtained from Cell Applications (San Diego, CA, USA). Human osteosarcoma HOS (RCB0992), MG63 (RCB1890), Saos-2 (RCB0428), 143B (RCB0701), and murine osteosarcoma LM8 (RCB1450) cells were purchased from Riken Cell Bank (Tsukuba, Japan). The cells were maintained in 10% FBS (GIBCO^®^, Life Technologies, Carlsbad, CA, USA) containing DMEM (GIBCO^®^, Life Technologies) (FBS/DMEM) supplemented with 100 U/mL penicillin and 100 μg/mL streptomycin at 37°C in a humidified atmosphere with 5% CO_2_.

### PAI Preparation

NTAPP was generated from helium using a PCT-DFJIM-02 model damage-free multi-gas plasma jet (Plasma Concept Tokyo, Tokyo, Japan), which generates a capacitively coupled plasma. The typical frequency was 20 kHz, with a peak voltage of 1 kV, current of 30 mA, and a helium flow rate of 3 L/min. PAI was made by irradiating plasma from above at a distance of 20 mm to 5 mL of Soldem 3A (TERUMO, Tokyo, Japan) for 1 or 5 min. The original PAI was diluted to a final concentration of 6.3–50% with 10% FBS/DMEM (for cell experiments) or Hank’s balanced salt solution (for biochemical experiments) and was indicated as PAI (6.3–50%). PAM was prepared as described above except for NTAPP irradiation to DMEM in place of Soldem 3A.

### Quantitation of H_2_O_2_ in PAI

According to the manufacturer, the concentration of H_2_O_2_ in PAI was measured using the Amplex Red Hydrogen Peroxide/Peroxidase Assay Kit (Thermo Fisher Scientific, Waltham, MA, USA) ‘s protocols. This assay is based on resorufin formation by the Amplex Red reagent reaction (10-acetyl-3, 7-dihydroxyphenoxazine) with H_2_O_2_ in a 1:1 stoichiometry in the presence of peroxidase. Briefly, samples were placed on a 96-well plate (50 mL/well), added with 50 μL of a working solution of 100 mM of Amplex Red reagent and 0.2 U/mL of horseradish peroxidase, and incubated at room temperature for 30 min. Absorbance at 570 nm was measured using a microplate reader (Nivo 3F Multimode Plate Reader, PerkinElmer, Waltham, MA, USA). The concentrations of H_2_O_2_ were calculated using a standard curve, prepared using authentic H_2_O_2_ included with the kit.

### Measurements of Intracellular ROS Generation

LM8 OS cells were cultured in 6-well plates for 24 h and then exposed to PAI (50%) or H_2_O_2_ (100 μM) for 1 h. Cells were labeled with DCFH-DA FITC antibody using the Reactive Oxygen Species (ROS) Detection Assay Kit obtained from BioVision, Inc. (Milpitas, CA, USA) according to the manufacturer’s instructions. Data were collected using a FACSCalibur (BD Biosciences, Franklin Lakes, NJ, USA) and were analyzed using CellQuest Pro (BD Biosciences) and FlowJo software (TreeStar, Ashland, OR, USA). Images of ROS formation in LM8 live cells in each well were taken with the Fluorescence Microscope FLUOVIEW FV10i (Olympus, Tokyo, Japan) (Ex/Em 495/529 nm). Experiments were performed in triplicate.

### Animals

Homozygous wild-type (WT) C3H/HeJJcl mice were purchased from CLEA Japan (Tokyo, Japan). The mice were housed at 22–24°C with a 12-h light/dark cycle with standard mouse chow and water provided *ad libitum*. All experiments with mice were conducted according to the Guidelines for Proper Conduct of Animal Experiments, Science Council of Japan, and the protocols were approved by the Animal Care and Use Committee (No. 17–11) at the University of Yamanashi.

### Cell Viability Assay

Cell viability was measured by the WST-8 assay using Cell Counting Reagent SF (Nacalai Tesque, Inc., Kyoto, Japan) or Cell Counting Kit-8 (Dojindo Molecular Technologies, Inc., Kumamoto, Japan) according to the manufacturer’s instructions. These methods are colorimetric assays based on the formation of a water-soluble formazan product (WST-8). Briefly, cells were seeded at a density of 8 × 10^3^ cells/well in 96-well plates (Corning, Inc., Corning, NY, USA) and cultured with agents to be tested for 72 h at 37°C before adding 10 μL cell counting reagent and further incubation for 2 h. Absorbance at 450 nm was measured using a microplate reader (Nivo 3F, PerkinElmer or SH-1100R (Lab), Corona Electric Co., Ltd., Ibaraki, Japan).

### Cell Death Assay

Cells were cultured in 6-well plates for 24 h and then exposed to several PAI concentrations for 24 to 48 h. The cells were retrieved using Versene (GIBCO^®^, Life Technologies) and incubated with Annexin V and 7AAD (BD Biosciences) for 15 min to evaluate apoptotic cell death. Data were collected using a FACSCalibur (BD Biosciences). Annexin V-negative and 7AAD-positive cells were defined as necrotic cells. Annexin V-positive and 7AAD-negative cells were defined as early apoptotic cells. Data obtained were analyzed by CellQuest Pro (BD Biosciences) and FlowJo software (TreeStar). Experiments were performed in triplicate. Whole-cell death was evaluated using a commercially available kit (Live/Dead Viability/Cytotoxicity Kit; Invitrogen, Carlsbad, CA, USA) according to the manufacturer’s instructions as described previously (33). Briefly, the cells were cultured on an 8-well imaging chamber (Imaging Chamber 8 CG, Zell-Kontakt GmbH, Nörten-Hardenberg, Germany) and treated with the agents for 24 h at 37°C. The cells were stained with 4 μM each of calcein-AM and ethidium bromide homodimer-1 (EthD-1) to label live cells in green and dead cells in red, respectively. Images were obtained using a BZ X-710 Digital Biological Microscope (Keyence Corporation, Osaka, Japan) equipped with a 40×, 0.60 numerical aperture (NA) LUCPlanFL N objective (Olympus) and were analyzed using BZ-H3A application software (Keyence Corporation).

### Tumor Growth and Metastasis

PAI or Sal’s ability to reduce tumor growth *in vivo* was evaluated using allograft transplants of LM8 murine OS cells in mice. Male C3H/HeJJcl mice of 8 weeks age were administered general anesthesia with isoflurane (ISOFLU^®^) (Abbott Laboratories, North Chicago, IL, USA) and oxygen. LM8 murine osteosarcoma cells (2 × 10^6^ cells/mouse) in 0.1 mL DMEM were injected subcutaneously into the back of the mice on day 0. Three times per week after day 7, 200 μL of PAI (50%) and Sal (3 mg/kg) alone or in combination was administered intravenously to 6 mice in each group. The mice were weighed, and the size of the primary tumors was measured weekly. On day 35, all mice were sacrificed (**Supplementary Figure 6A**). Tumors and lungs of mice were fixed in 10% formalin neutral buffer solution for 3 days. Tumors and lungs were paraffin-embedded, and consecutive 5-μm sections were stained with hematoxylin-eosin purchased from Merck.

### Immunohistochemical (IHC) Staining

IHC staining was performed as previously described (45). IHC staining with primary antibodies against LC3-I/II (D3U4C) (#12741, 1:2000), Beclin-1 (D40C5) (#3495, 1:400) (Cell Signaling Technology, Danvers, MA, USA) and Ki67 (SP6) (ab16667, 1:100) (Abcam, Cambridge, UK) was performed using the Dako Liquid DAB+Substrate Chromogen System (Glostrup, Denmark) according to the manufacturer’s specifications, followed by counterstaining with hematoxylin.

### Autophagy Assay

Cells were cultured in 6-well plates for 24 h and then exposed to several PAI concentrations for 24–48 h. According to the manufacturer’s instructions, the cells were stained and analyzed using the Cyto-ID^®^ Autophagy Detection Kit obtained from Enzo Life Sciences. Data were collected using a FACSCalibur (BD Biosciences) and analyzed using CellQuest Pro (BD Biosciences) and FlowJo software (TreeStar). Experiments were performed in triplicate.

### Western Blotting Analysis

Cells were washed in Ca^2+^-, Mg^2+^-free PBS, lysed during 15 min shaking in CelLytic M lysis buffer (Merck KGaA) with a protease inhibitor cocktail and phosphatase inhibitor cocktail (both from Sigma). Protein concentrations were determined using a BCA protein assay (Thermo Fisher Scientific) according to the manufacturer’s protocol. Equal amounts of protein from each sample were analyzed by immunoblotting with primary antibodies against cleaved caspase-3 (Asp175) (5A1E) (#9664, 1:1000), cleaved caspase-8 (Asp387) (D5B2) (#8592, 1:1000), phospho-RIP1 (Ser166) (#31122, 1:1000), RIP (D94C12) (#3493, 1:1000), phospho-P70-S6 (Thr389) (108D2) (#9234, 1:1000), P70-S6 (49D7) (#2708, 1:1000), phospho-Rictor (Thr1135) (D30A3) (#3806, 1:1000), Rictor (53A2) (#2114, 1:1000), phospho-Raptor (Ser792) (#2083, 1:1000), LC3-I/II (D3U4C) (#12741, 1:1000), and GAPDH (D16H11) (#5174, 1:1000) obtained from Cell Signaling Technology; phospho-RIP3 (phospho S232) (EPR9516(N)-25) (ab195117, 1:1000), Raptor (EP539Y) (ab40768, 1:500) obtained from Abcam; and phospho-P62 (Ser351) (SQSTM1) (PM074, 1:500) and P62 (SQSTM1) (PM045, 1:1000) obtained from MBLI (Woburn, MA, USA), were used. Images were captured using a LAS-4000 camera system from Fujifilm (Tokyo, Japan).

### Live-Cell Mitochondrial Network Imaging

The mitochondrial network in live cells was analyzed as previously described (11). Briefly, cells in FBS/DMEM (3 × 10^4^/well) adherent on 8-well chambered coverslips were treated with the agents to be tested for 24 h at 37°C in a 5% CO_2_ incubator. After removing the medium by aspiration, the cells were washed with fresh FBS/DMEM and stained with 20 nM MitoTracker Red CMXRos for 1 h at 37°C in the dark in a 5% CO_2_ incubator. In some experiments, the nuclei were counterstained with 1 mg/mL of Hoechst 33342. The cells were then washed with and immersed in FluoroBrite™ DMEM (Thermo Fisher Scientific). Images were obtained using a BZ X-710 Digital Biological Microscope (Keyence) equipped with a 100 ×, 1.40 n.a. UPlanSApo Super-Apochromat, coverslip-corrected oil objective (Olympus). Images were analyzed using BZ-H3A application software (Keyence) and ImageJ software (NIH, Bethesda, MD, USA). For each sample, the morphology of mitochondria in 30–60 cells was analyzed, and the percentages of four different types, i.e., tubular/fused, fragmented, and swollen/clustered, were calculated.

### Statistical Analysis

Data are presented as mean ± standard deviation (SD) or standard error (SE) and were analyzed by one-way analysis of variance followed by Tukey’s *post hoc* test using add-in software with Excel 2016 for Windows (SSRI, Tokyo, Japan). For some experiments, significance was determined using Student’s *t*-test after an F-test. *P <* 0.05 was considered statistically significant.

## Results

### PAI Contains Oxidants and Evokes the Subsequent Intracellular ROS Generation

**Figure 1:**
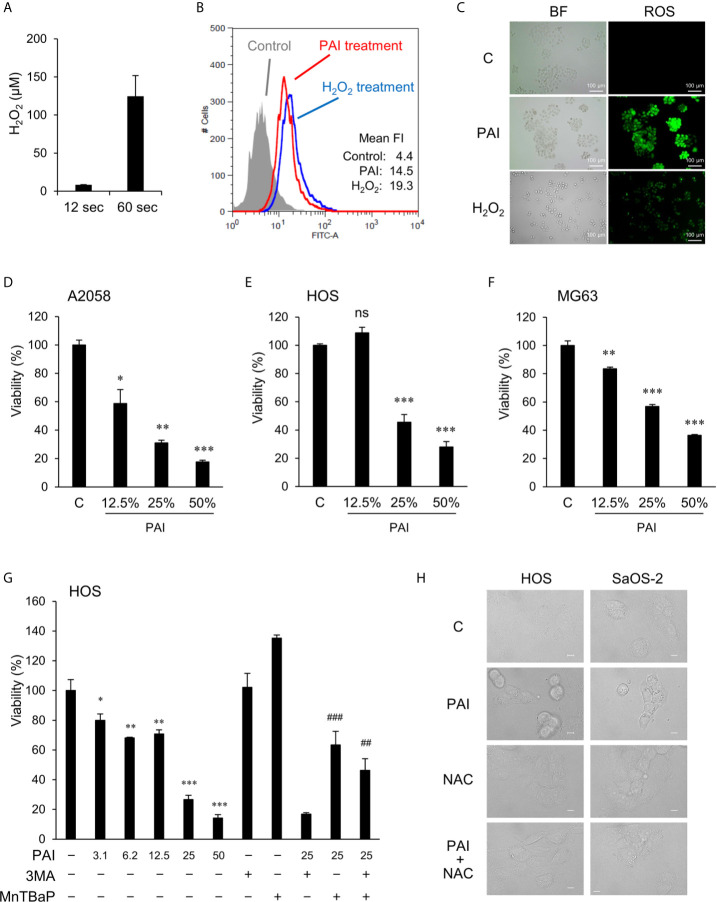
PAI reduces cell viability in MM and OS cells in a ROS-dependent manner. **(A)** Measurements of H_2_O_2_ in PAI. Non-thermal atmospheric pressure helium plasma was irradiated from above at a distance of 20 mm to 5 mL of the infusion fluid Soldem 3A in a 6-well plate for 1 or 5 min (12 or 60 s/mL). Then, H_2_O_2_ concentration in PAI was quantitated using the Amplex Red reagent. Data are the mean ± SD (n = 3). **(B, C)** Analysis of intracellular ROS generation in live cells. LM8 OS cells (1 × 10^6^) were labeled with DCFH-DA, treated with PAI (50%) or H_2_O_2_ (100 μM), and analyzed by **(B)** flow cytometry and **(C)** fluorescence microscopy. Histograms in **(B)** represent fluorescence intensity (FI), and green fluorescence in **(C)** shows ROS production in the cells treated with PAI and H_2_O_2_. **(D–F)** PAI reduces cell viability in MM and OS cells. **(D)** A2058, **(E)** HOS, and **(F)** MG63 cells were treated with the indicated PAI concentrations for 72 h and analyzed for viability using the WST-8 cell growth assay. Data are the mean ± SD (n = 3). Data were analyzed by one-way analysis of variance followed by Tukey’s *post hoc* test. *P < 0.05; **P < 0.01, ***P < 0.001; n.s., not significant, vs. control treated with vehicle. **(G)** Effect of 3-MA and MnTBaP on reduced cell viability. HOS cells were treated with the indicated concentrations of PAI alone or in combination with 3-MA (1.3 mM) or MnTBaP (30 μM) for 72 h and were analyzed for viability as described above. Data are the mean ± SD (n = 3). *P < 0.05; **P < 0.01; ***P < 0.001 vs. control treated with vehicle. ^##^ *P <* 0.05 vs. PAI + 3-MA; ^###^ *P <* 0.01 vs. PAI alone. **(H)** HOS and Saos-2 cells were treated with PAI (25%) or *N*-acetylcysteine (2 mM) alone or in combination for 18 h and observed under a BZX-710 digital biological microscope with a 100× coverslip-corrected oil objective and were analyzed using BZ-H3A application software. Bar = 10 μm.

Various reactive oxygen/nitrogen species (ROS/RNS) are generated in the air, and liquid phases following NTAPP irradiation and relatively long-lived species are thought to be responsible for the anticancer activity (1, 5, 6). H_2_O_2_ is found in various types of PALs and was shown to be the primary mediator of their anticancer activity (4–8). Therefore, we first quantitated the amounts of H_2_O_2_ in PAI. We previously found that PAM’s anticancer activity was correlated with the time of NTAPP irradiation and inversely correlated with the volume of the target solution (11). Accordingly, the ratio of time and volume is critical for determining efficacy. As expected, PAI contained substantial amounts of H_2_O_2_, which increased as the ratio increased (**Figure 1A**); the amounts were 8.37 ± 0.29 and 124.6 ± 27.1 (μM) (n = 3) for NTAPP irradiation of 12 s (0.2 min/mL) and 60 s (1 min/mL), respectively. Previously, we demonstrated that PAM stimulates intracellular ROS generation, including mitochondrial superoxide (11). Flow cytometric analysis showed that, like PAM, PAI treatment increased the intracellular ROS level in LM8 OS cells (**Figure 1B**). We also detected robust ROS generation in live adherent cells (**Figure 1C**). In this case, a much stronger ROS signal was observed with PAI than with H_2_O_2_ (100 μM). These results indicate that PAI contains H_2_O_2_ and can evoke subsequent intracellular ROS generation.

### PAI Reduces Cell Viability in MM and OS Cells in a ROS-Dependent Manner

**Figure 2:**
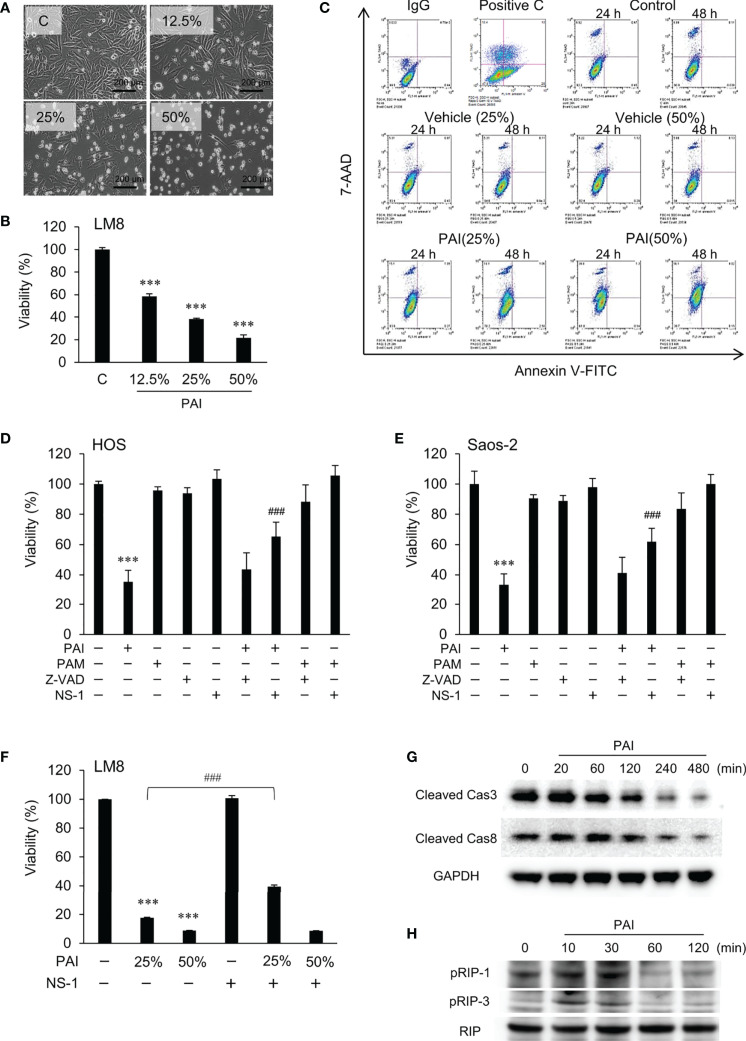
PAI induces necroptosis. **(A)** LM8 cells were treated with varying concentrations of PAI for 18 h and observed by light microscopy. Bar = 200 μm. The cells became less adherent, shrunken, and round in a dose-dependent manner. **(B)** LM8 (5 × 10^3^ cells) were treated with the indicated PAI concentrations for 72 h and analyzed for viability using the WST-8 cell growth assay. Data are the mean ± SD (n = 4). Data were analyzed by one-way analysis of variance followed by Tukey’s *post hoc* test. ***P < 0.001 vs. control treated with vehicle. **(C)** Cells were treated with PAI (25% or 50%) and the vehicle for 24 or 48 h, stained with Annexin V-FITC and 7-AAD, and analyzed by flow cytometry. Gemcitabine was used as a positive control for apoptosis induction (Positive C). The rate of cell necrosis and cell apoptosis is shown in **Supplementary Figures 1A, B**. **(D–F) (D)** HOS, **(E)** Saos-2 or **(F)** LM8 cells were treated with PAI (25% or 50%) or PAM (25%) alone or in combination with ZVAD-FMK (ZVAD, 10 μM) or necrostatin-1 (NS-1, 30 μM) for 72 h and cell viability was measured by the WST-8 assay. Data are the mean ± SD (n = 3). Data were analyzed by one-way analysis of variance followed by Tukey’s *post hoc* test. ***P < 0.001 vs. control. ^###^ *P <* 0.001 vs. PAI alone. **(G, H)** The status of Caspase-3, Caspase-8, RIPK1, and RIPK3 activity in the cells. LM8 cells were treated with PAI (50%) for the indicated time and then analyzed for the expression of cleaved caspase-3, cleaved caspase-8, phosphorylated RIP-1, and phosphorylated RIP-3 by western blotting analysis using specific antibodies. GAPDH and RIP were used as the loading control. See **Supplementary Figures 1C, D**, **2A, B** for examples of uncropped images and quantification for each antibody.

We examined the ability of PAI to reduce cell viability. Cells were treated with varying PAI concentrations for 72 h and analyzed for their viability in a WST-8 cell growth assay. PAI treatment resulted in a dose-dependent decrease in the viability of A2058 MM, HOS, and MG63 OS cells (**Figures 1D–F**) compared to control cells treated with the vehicle (infusion fluid). PAI was also effective in other OS cell lines, including LM8 and Saos-2 (**Figure 2**) and 143B cells (**Figure 4**). We previously reported that ROS (11) and autophagic cell death (43) mediated PAM’s anticancer effect. Therefore, we examined the possible roles of these events in the effects of PAI. The superoxide dismutase mimetic MnTBaP significantly inhibited cell death, whereas the autophagy inhibitor 3-MA did not (**Figure 1G**). Moreover, the broad-spectrum antioxidant *N*-acetylcysteine completely prevented PAI-induced cell damage in HOS and Saos-2 cells (**Figure 1H**). These results suggest that ROS, but not autophagy, plays a role in mediating cell death induced by PAI.

### PAI Induces Necroptosis

To determine the cell death modality, we observed cells’ morphology following PAI treatment under a light microscope. **Figure 2A** shows the results obtained in LM8 cells. The adherent spindle cells were converted to less adherent, shrunken, and round cells in a dose-dependent manner. These morphological changes were associated with a dose-dependent decrease in cell viability (**Figure 2B**). Double staining with FITC-conjugated Annexin V and 7-AAD followed by flow cytometric analyses showed a dose- and time-dependent increase in Annexin V-negative, 7-AAD-positive cells and minimal increase in Annexin V-positive cells with PAI treatment (**Figure 2C** and **Supplementary Figures 1A, B**). These results suggest that PAI preliminarily causes necrotic cell death and that apoptosis plays a minor role. To confirm this, we examined the effect of pharmacological inhibitors specific for apoptosis and necroptosis. Receptor interacting protein (RIP) kinases 1 and 3 have essential scaffolding functions that activate necroptosis in cells. In HOS and Saos-2 cells, necrostatin-1 (NS-1), a specific inhibitor of RIP1 kinase (RIPK), inhibited cell death significantly, whereas the broad-spectrum caspase inhibitor Z-VAD-FMK (ZVAD) had minimal effects (**Figures 2D, E**). Additionally, NS-1 significantly reduced PAI-induced cell death in LM8 cells treated with PAI (25%) but not PAI (50%) (**Figure 2F**). Next, we examined the status of caspase-3, caspase-8, RIPK1, and RIPK3 activity in the cells. Western blot analysis showed that the expression of activated caspase-3 and caspase-8 (cleaved caspase-3 and caspase-8) were decreased following PAI treatment (**Figure 2G** and **Supplementary Figures 1C, D**). In contrast, expression of activated RIPK1 and RIPK3 (phosphorylated-RIPK1 and -RIPK3) was increased (**Figure 2H** and **Supplementary Figures 2A, B**). This effect was rapid and transient; the increases were initially observed within 10 min, reached a maximum after that, and declined to the basal levels by 60 min. These results indicate that PAI primarily induces necroptosis in OS cells.

### PAI Reduces Autophagic Flux by Promoting the mTORC Pathway

**Figure 3:**
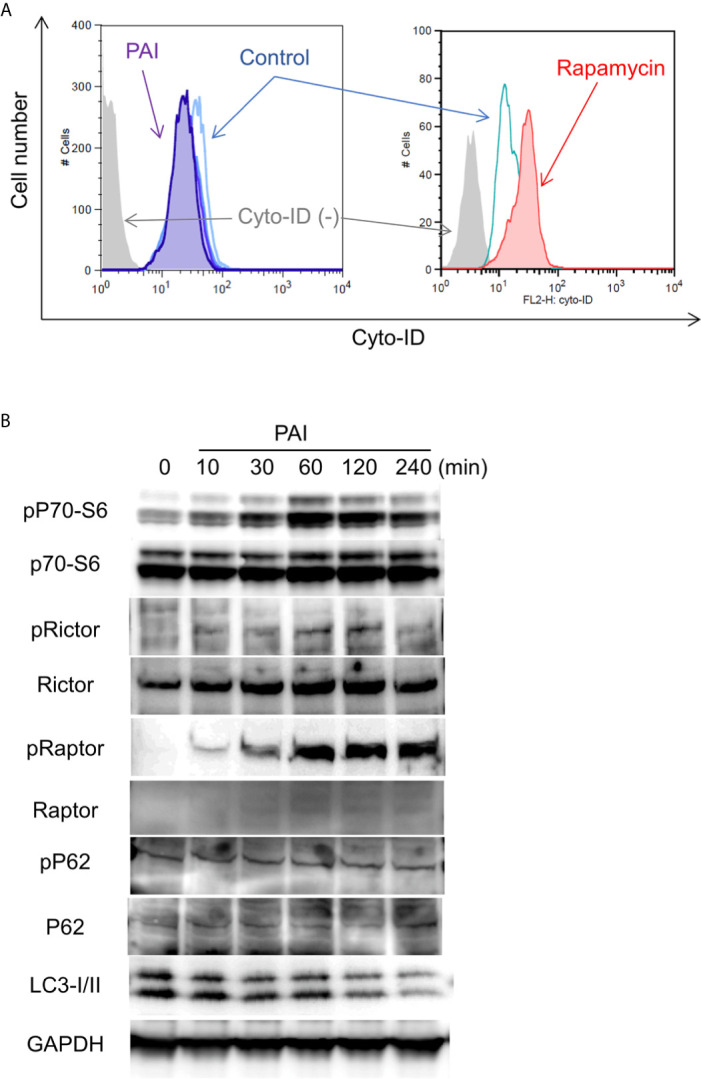
PAI reduces autophagic flux by promoting the mTOR complex pathways. **(A)** LM8 cells were loaded with Cyto-ID and treated with PAI (50%) or rapamycin (positive control) for 18 h and analyzed by flow cytometry. **(B)** LM8 cells were treated with PAI (50%) for the indicated time and then analyzed for the expression of p70-S6 kinase, Rictor, Raptor, p62, and LC3-I/II, and their phosphorylated forms by western blotting analysis using specific antibodies. GAPDH was used as a loading control. See **Supplementary Figures 3**, **4** for examples of uncropped images and quantification for each antibody.

The results shown in **Figure 1** suggest that autophagic cell death plays a minor role in the anticancer effect of PAI. In cancers, autophagy exerts both cytocidal and cytoprotective roles. In support of this view, we previously reported that human MM and OS cells exhibit a substantial level of autophagic flux even under stress-free and nutritional conditions and that ambient autophagy prevented the cells from spontaneous and TRAIL-induced apoptosis (43, 44). Therefore, next, we determined the possible role of cytoprotective autophagy. We investigated the effect of PAI on the autophagic flux in OS cells. When Cyto-ID, the specific probe of autophagosome formation, was analyzed by flow cytometry, rapamycin treatment substantially increased the signal, whereas PAI significantly reduced it (**Figure 3A**). mTORC plays a pivotal role in regulating autophagy by inhibiting phagophore formation (15, 18, 19). To determine whether this complex participates in the suppression of autophagy, we examined the effect of PAI on mTORC activation by western blotting analysis. The results showed that PAI increased the phosphorylation of p70-S6 kinase, the substrate of mTOR kinase1/2, and the phosphorylation of Rictor and Raptor, which are components of these two kinases. PAI decreased the protein levels of LC3-I/II, an autophagosomal marker, over time (**Figure 3B** and **Supplementary Figures 3**, **4**).

### PAI and Sal Mutually Act as an Adjuvant in a Tumor-Specific Manner

**Figure 4:**
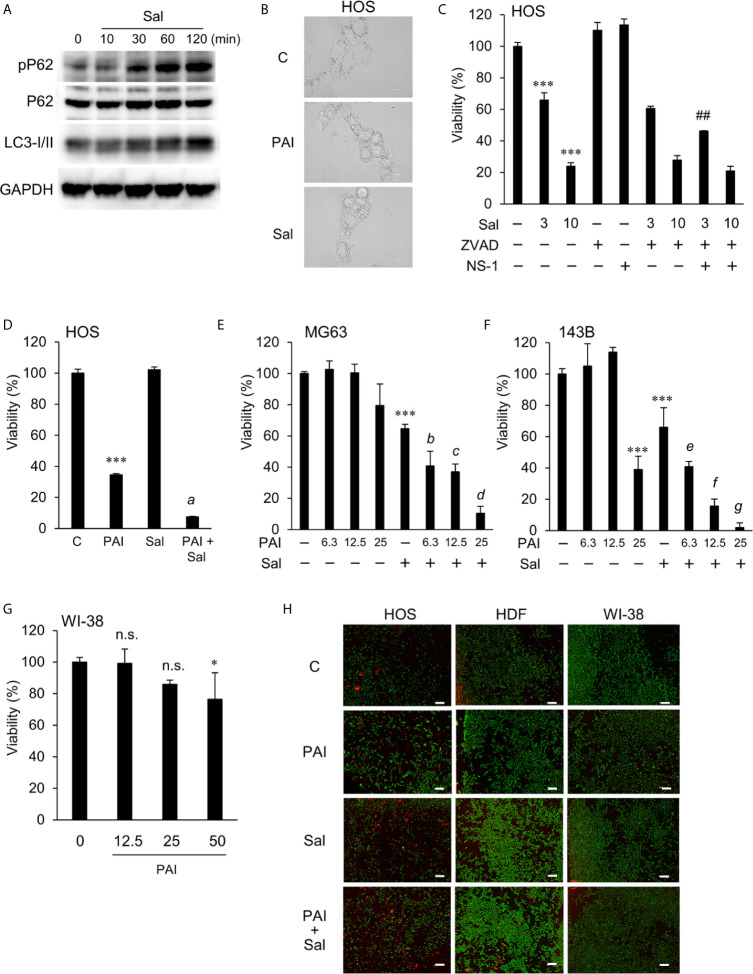
PAI and Sal mutually act as adjuvant in a tumor-specific manner. **(A)** LM8 cells were treated with Sal (1 μM) for the indicated time and then analyzed for the expression of phosphorylated P62, P62, and LC3-I/II by western blotting analysis using specific antibodies. GAPDH was used as the loading control. See **Supplementary Figure 5** for examples of uncropped images and quantification for each antibody. **(B)** HOS cells were treated with PAI (25%) and Sal (3 μM) for 18 h and observed under a BZX-710 digital biological microscope equipped with a 100×coverslip-corrected oil objective and were analyzed using BZ-H3A application software. Bar = 10 μm. **(C)** HOS cells were treated with Sal (3, 10 μM) alone or in combination with ZVAD-FMK (ZVAD, 10 μM) or necrostatin-1 (NS-1, 30 μM) for 72 h, and were measured for cell viability using the WST-8 assay. **(D)** HOS cells were treated with PAI (25%) and Sal (1 μM) alone or in combination for 72 h and were measured for cell viability using the WST-8 assay. Data were analyzed by one-way analysis of variance followed by Tukey’s *post hoc* test. ***P < 0.001 vs. control; *a*, *P <* 0.001 vs. PAI or Sal alone. **(E, F) (E)** MG63 and **(F)** 143B cells were treated with PAI at the indicated concentrations and Sal (3 μM) alone or in combination for 72 h and were measured for cell viability using the WST-8 assay. ***P < 0.001 vs. control. *b*, *P < 0.05; *c*, ***P < 0.001; vs. Sal alone. *e*, *P < 0.05; *f, g*, ***P < 0.001; vs. Sal alone. **(G)** WI-38 cells were treated with PAI at the indicated concentrations for 72 h and were measured for cell viability using the WST-8 assay. *P < 0.05; n.s., not significant, vs. control. **(H)** HOS, human dermal fibroblasts, and WI-38 cells were treated with PAI (25%) and Sal (3 μM) alone or in combination for 18 h and were measured for calcein-AM and ethidium bromide homodimer-1 (EthD-1) staining. Live cells were stained green with calcein, whereas dead/damaged cells were stained red with EthD-1. Color images were obtained using a BZX-710 digital biological microscope equipped with a 40× objective and were analyzed using the BZ-H3A application software. Zoomed images represent the magnification of the representative images in the box. Bar = 300 μm. Cells positive for EthD-1 were counted and quantified in **Supplementary Figure 6**. ^##^ *P <* 0.01 vs. Sal + ZVAD.

Although the mechanisms by which Sal exhibited the anticancer effect remain unknown, recent studies demonstrated that Sal targets autophagy (26–35). Therefore, we determined the effect of Sal on autophagy flux. Western blot analysis showed that Sal treatment resulted in increased phosphorylation of p62 (phospho-p62) and LC3-I/II in LM8 cells (**Figure 4A** and **Supplementary Figure 5**). These increases were observed as rapidly as 30 min following Sal treatment and became more evident over time up to 120 min. Thus, Sal promotes apoptosis in cells. Next, we examined whether Sal could induce cell death in our cell systems. As shown in **Figure 4B**, Sal induced cell death, which was characterized by an increase in the number of detached and round cells along with membrane blebbing. Thus, the effect resembled that caused by PAI. Sal at concentrations of ≥3 μM significantly reduced cell viability, and Z-VAD-FMK minimally inhibited this effect while Z-VAD-FMK plus NS-1 partially blocked it (**Figure 4C**). Combined application of PAI and Sal appeared to cause more severe cell damage than either agent alone. To confirm this cooperative action, we examined the combined effect of PAI and Sal treatment on cell viability. We found that 3 μM was a borderline range in which Sal had a substantial or modest cytotoxic effect depending on cell types. When used with PAI, a greater extent of cell death was reproducibly observed than that caused by either single agent (**Figure 4D**). This effect was observed over a wide range of PAI (6.3–50%) in different OS cell lines (**Figures 4E, F**). In contrast, treatment with PAI (12.5–50%) for 72 h only modestly decreased the viability of WI-38 cells (maximum of 24% at 50%) (**Figure 4G**). Staining with calcein-AM and EthD-1 showed that treatment with PAI and Sal alone or in combination for 18 h substantially increased the proportion of EthD-1-positive (dead/damaged) cells in HOS cells along with decreased calcein-positive (live) cells. In contrast, they minimally increased the proportion of PI-positive cells and decreased the proportion of calcein-positive cells in human dermal and WI-38 lung fibroblasts (**Figure 4H** and **Supplementary Figure 6**).

### Combined Administration of PAI and Sal Reduces the Growth and Metastasis of OS Allografts With Minimal Adverse Effects

**Figure 5:**
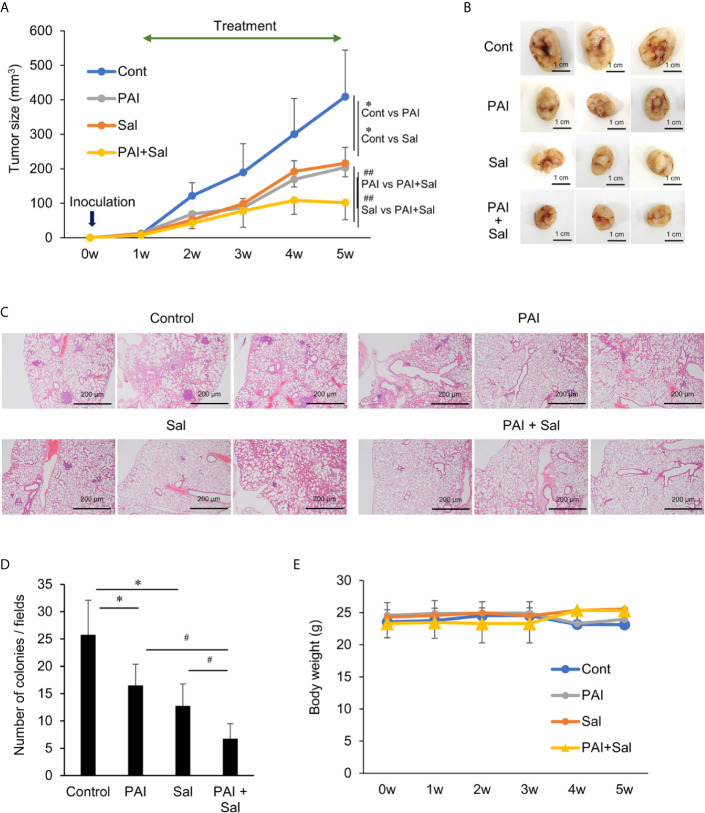
Combined administration of PAI and Sal reduces the growth and metastasis of the murine OS Allograft model. **(A)** C3H mice were subcutaneously inoculated with LM8 cells (2 × 10^6^) at day 0 and intravenously administered 200 μL of PAI (50%) and Sal (3 mg/Kg) alone or in combination 3 times per week for 4 weeks from day 7. The sizes of the tumors in the mice were measured every week. Values represent the mean ± SD (n = 6). *P < 0.05 compared with the corresponding control. ^##^ *P* < 0.01 vs. PAI or Sal alone. **(B)** Representative photos of primary tumors at 5 weeks after inoculation of LM8 cells. Bar = 1 cm. Simultaneously, lungs were removed, and metastatic nodules on their surface were counted with a stereomicroscope. **(C)** Representative microscope image of lungs (hematoxylin-eosin staining). Bar = 200 μm. **(D)** Quantitative analysis of metastatic nodules in the lungs. Data were analyzed by one-way analysis of variance followed by Tukey’s *post hoc* test. Results are shown as mean ± SD. *P < 0.05 compared with the corresponding control. ^#^ *P* < 0.05 vs. PAI or Sal alone. **(E)** The weights of the mice were measured every week for 5 weeks. Data are the mean ± SD (n = 6). See **Supplementary Figure 7** schematic flowchart and photos of the tumor-bearing mouse.

**Figure 6:**
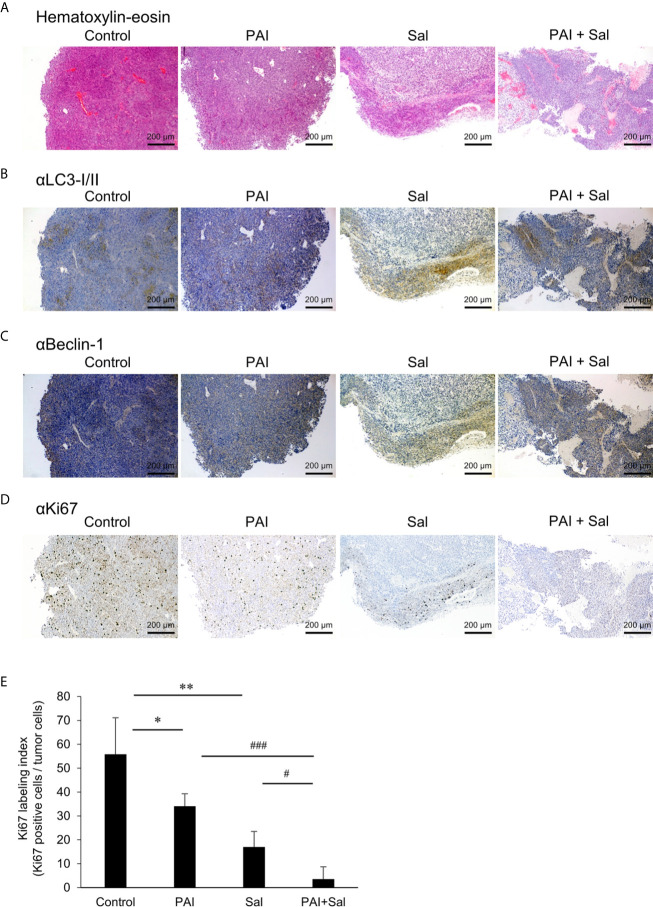
Combined administration of PAI and Sal reduced the Ki67 labeling index. **(A–E)** Primary tumors were resected at day 35 after inoculation of LM8 cells. Hematoxylin-eosin staining **(A)** and IHC staining with primary Abs against LC3-I/II **(B)**, Beclin-1 **(C)**, and Ki67 **(D)** were performed. **(E)** Quantitative analysis of metastatic nodules in the lungs. Data were analyzed by one-way analysis of variance followed by Tukey’s *post hoc* test. Results are shown as mean ± SD. *P < 0.05; **P < 0.01 compared with the corresponding control. ^#^ *P <* 0.05; ^###^ *P <* 0.001 vs. PAI or Sal alone.

Next, we determined whether PAI and Sal operate cooperatively *in vivo.* LM8 can be transplanted into C3H mice to induce the development of tumors with high metastatic potential to the lung after inoculation into the skin. We assessed the anticancer activity of PAI and Sal alone or in combination in this allograft model (**Supplementary Figure 6A**). After subcutaneous inoculation of LM8 cells into mice, tumors exhibited rapid growth and reached a size of about 400 mm^3^ within 5 weeks. Treatment of the mice by intravenous administration of PAI and Sal 3 times weekly resulted in a significant (47.2% and 50.2%, respectively) reduction in the primary tumor size (**Figures 5A, B** and **Supplementary Figure 6B**). A more pronounced anticancer effect (75.1% reduction) was observed in mice treated by combined administration of PAI and Sal (**Figures 5A, B**). Additionally, histological analysis with hematoxylin-eosin stain revealed that PAI and Sal significantly decreased the colony number of metastatic nodules in the lungs of LM8-inoculated mice. Again, combined administration of PAI and Sal was more effective than a single administration of either agent (**Figures 5C, D**). Despite the potent anticancer effect, there was a minimal difference in weight between the mice in the treated and untreated groups, and no obvious abnormality was observed in treated mice with this protocol (**Figure 5E**). These results indicate that combined administration of PAI and Sal has potent and tumor-selective anticancer effects *in vivo*. The impact of PAI and Sal on autophagy *in vivo* was determined by IHC analyses using specific antibodies against LC3-I/II and Beclin-1. LC3-I//II and Beclin-1 levels were lower in the PAI treatment group and higher in the Sal treatment group. These levels in the combined administration of PAI and Sal group were intermediate (**Figures 6A–C**). The tumor proliferation index marker Ki67 is intimately associated with tumor cell proliferation (46, 47). The Ki67 labeling index for the PAI or Sal administration group was significantly lower than that of the control group. Furthermore, combined treatment with these agents reduced this index more significantly than each monotherapy (**Figures 6D, E**). these results are consistent with with our *in vitro* study.

### PAI and Sal Alter Mitochondrial Morphology Cooperatively

**Figure 7:**
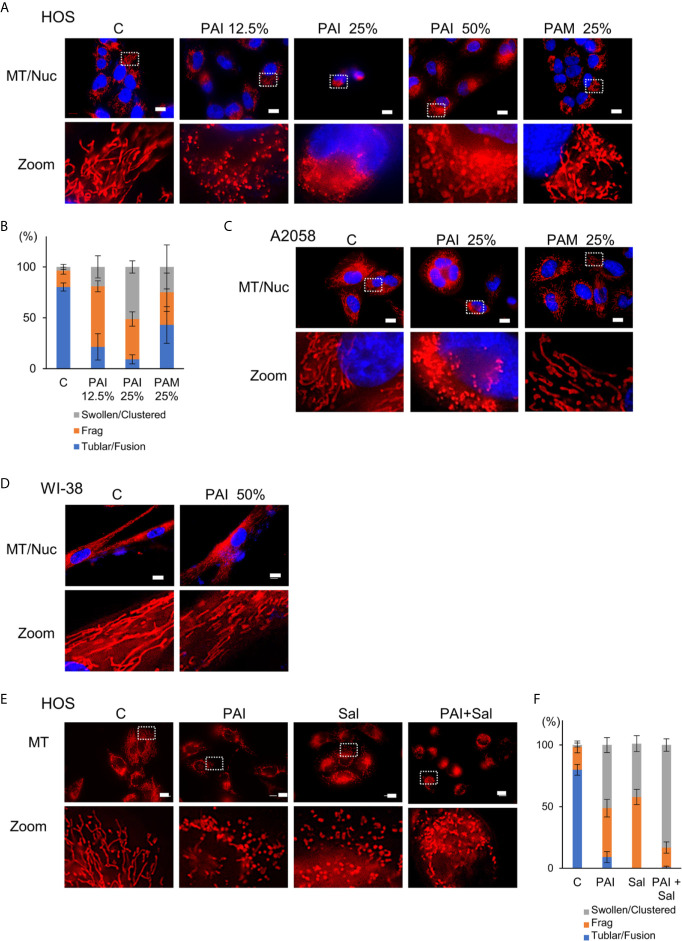
Combined administration of PAI and Sal induces mitochondrial network collapse in a tumor-selective manner. **(A, E)** HOS, **(C)** A2058 cells, and **(D)** WI-38 fibroblasts grown on coverslips were treated with PAI at the indicated concentrations and PAM (25%) **(A)**, with PAI (25%) and PAM (25%) **(C)**, with PAI (50%), **(D)** or with PAI (25%) and Sal (1 μM) alone or in combination **(E)** for 18 h. After washing, the cells were stained with MitoTracker Red CMXRos (for mitochondria, MT) for 1 h and washed again. The nuclei were counterstained with Hoechst 33342 (Nuc). Images were obtained using a BZX-710 digital biological microscope equipped with a 100×coverslip-corrected oil objective and were analyzed using BZ-H3A application software. Zoom images represent the magnification of the representative images in the box. Bar = 10 μm. **(B, F)** Quantitative analysis of mitochondrial morphology in the cells. For each sample, the morphology of mitochondria in 30~60 cells was analyzed, and the percentages of four different types, i.e., tubular/fused, fragmented, and swollen/clustered, were calculated. Data in six independent experiments were analyzed by one-way analysis of variance followed by Tukey’s *post hoc* test. Results are shown as mean ± SD.

Previously, we demonstrated that PAM induced excessive mitochondrial fragmentation along with swelling and clustering in tumor cells. In contrast, mitochondrial morphology was minimally changed in non-transformed cells following PAM treatment (11). Therefore, we examined whether PAI also affects mitochondrial morphology in cancer cells. We stained the mitochondria in live cells with MitoTracker Red and observed them under a biological microscope. The nuclei were counterstained with Hoechst 33342. Control cells were highly adherent and spindle cells, in which most mitochondria displayed a reticular network around the intact nuclei (**Figure 7A**, left panels). PAI dose-dependently altered mitochondrial morphology. Upon treatment with PAI (12.5%), the mitochondria were fragmented, and some became swollen (**Figure 7A**, second panels from left, **Figure 7B**). At higher PAI concentrations (≥25%), most mitochondria became fragmented, swollen, and clustered at the perinuclear regions of one side of the nuclei (**Figure 7A**, third and fourth panels from left, **Figure 7B**). These changes were pronounced in less adherent, round, and blebbing cells. PAM was less effective than PAI in modifying mitochondrial morphology. PAM (25%) led to only modest mitochondrial fragmentation with little swelling (**Figure 7A**, right panels). We obtained similar results in A2058 cells (**Figure 7C**). In contrast, even the highest PAI concentration (50%) led only to modest mitochondrial fission in WI-38 fibroblasts with minimal swelling and clustering (**Figure 7D**). We also examined the possible impact of Sal on mitochondrial morphology. Upon treatment with Sal (3 μM), the mitochondria underwent massive fragmentation, part of which was swollen in HOS cells (**Figure 7E**, third panels from left, **Figure 7F**). When administered together, PAI (25%) and Sal (1 μM) induced mitochondrial fragmentation with robust swelling and clustering in the cells (**Figure 7D**, right panels, **Figure 7F**).

## Discussion

In the present study, we showed that PAI had potent anticancer activity against MM and OS cells by inducing cell death (**Figures 1**, **2**, **4**) and reduced OS tumor growth and metastasis to the lung *in vivo* (**Figures 5**, **6**). Notably, PAI showed minimal cytotoxicity in non-transformed human dermal and lung fibroblasts (**Figure 4**), indicating that it preferentially injures tumor cells. PAI administration displayed no significant adverse effects, including weight loss *in vivo* (**Figure 5**), supporting its tumor-selective action. The antioxidant MnTBaP and *N*-acetylcysteine significantly inhibited the anticancer effect of PAI (**Figure 1**), suggesting that, like other PALs, PAI elicits the effect in a ROS-dependent manner. Consistent with this view, PAI contained H_2_O_2_ and increased the intracellular ROS (**Figure 1**). Our data indicated that unlike other PALs, including PAM, which mainly stimulate apoptosis (8–10), PAI primarily increased necrotic cell death, which was minimally affected by caspase inhibition (**Figure 2**). In support of this, PAI triggered necroptotic signaling pathways by rapidly and transiently activating RIP1 and RIP3 kinases (**Figure 2**). Moreover, the necroptosis-specific inhibitor NS-1 significantly but not completely reduced cell death caused by PAI (**Figure 2**). These data suggest that PAI primarily targets necroptosis. This finding is consistent with the observation that ROS plays a critical role in regulating necroptosis (48) and the cytotoxic effect of PAI (**Figures 1**, **2**, **4**). Although the reason for the limited effect of NS-1 is unknown, there are several possibilities. The simplest possibility is that another mode of programmed cell death mediates the effect of PAI. Alternatively, RIP1 and RIP3 kinases may be equally essential for transducing necroptotic cell death signaling. This idea is consistent with observations that PAI activated both kinases with similar kinetics (**Figure 2**). If both kinases were involved, it is logical that NS-1, which targets RIP1 signaling alone, failed to block the death signaling entirely. However, we did not observe complete inhibition of the anticancer effect of PAI even when the expression of the RIP1 and RIP3 genes was downregulated using specific siRNAs (data not shown). Thus, inhibition of necroptosis may lead to the onset of another mode of programmed cell death. Further investigation is necessary to clarify the role of another cell death mechanism in the anticancer effect of PAI.

Aggressive tumors have varying cellular machinery that protects them from apoptosis caused by anticancer agents, thereby conferring drug-resistance. An emerging view is that apoptosis (type I cell death) is not the sole mode of programmed cell death, but several different modes of cell death occur in response to anticancer agents. Of these, autophagy (type II cell death), paraptosis (type III cell death), and necroptosis are known. Thus, cancer therapy based on induction of non-apoptosis has been recently considered an alternative approach to treat apoptosis-resistant cancer cells, including MM and OS cells. We previously showed that PAM could induce autophagic cell death in MM and OS cells (43). PAM can increase the Cyto-ID signal and protein level of LC3-I/II, and the autophagy inhibitor 3-MA and bafilomycin A1 can block cell death in these cells. The present study showed that PAI reduced the ambient Cyto-ID signal and LC3-I/II protein expression (**Figure 3**). Moreover, 3-MA minimally affected cell death caused by PAI (**Figure 1**). These data indicate that autophagic cell death plays a minor role in the anticancer effect. In support of autophagy suppression, PAI can activate mTORC1/2, the autophagy suppressive machinery; further, PAI increased the phosphorylation of Raptor, Rictor, and their substrate P70-S6 kinase (**Figure 4**). At present, the mechanisms by which PAI activates these pathways are unknown. However, ROS may play a role in this context because mTORC activation is regulated by oxidative stress through a redox-sensitive mechanism (49–51). Autophagy contributes to cancer cell survival and resistance to different types of anticancer drugs (36–42). Thus, the suppression of autophagy may also participate in the anticancer effect of PAI.

**Figure 8:**
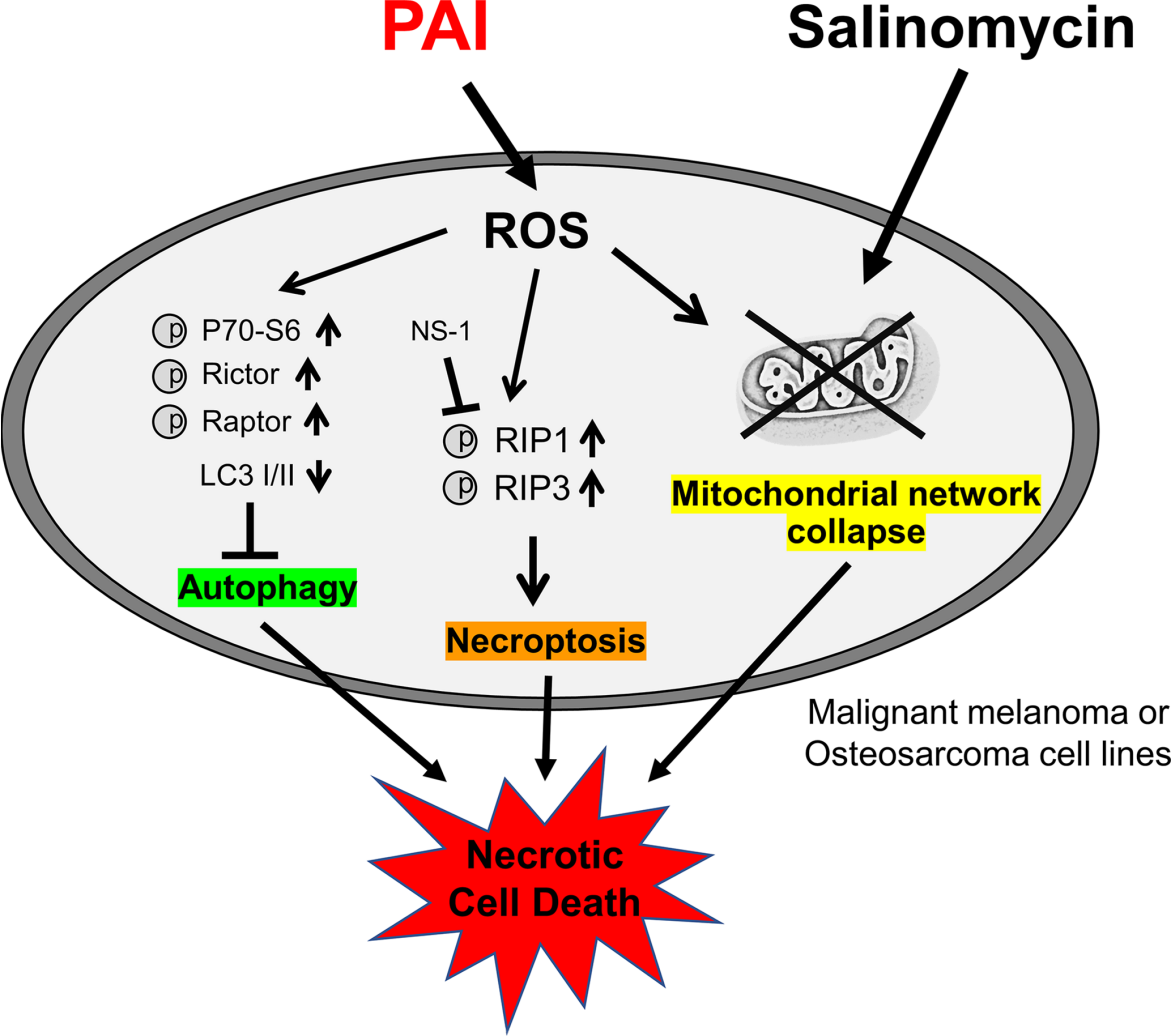
Graphical abstract of this study. PAI evokes necroptosis by RIP1/3 kinase activation while suppressing cytoprotective autophagy *via* mTORC 1/2 activation, and ROS may play a role in these effects. PAI also causes mitochondrial network collapse and dysfunction *via* ROS generation. Salinomycin may enhance mitochondrial aberration. All these events could contribute to the anticancer effect of PAI *via* necrotic cell death induction.

PAI and Sal mutually acted as adjuvant *in vitro* (**Figure 4**). The combination reduced tumor growth and metastasis to the lung while causing minimal adverse effects, including weight loss (**Figure 5**). The mechanisms underlying the cooperative actions between PAI and Sal are currently unknown. Notably, these two agents cooperatively disrupt the mitochondrial network. An emerging view is that mitochondrial network homeostasis plays a crucial role in regulating cancer cell survival, thereby serving as a promising target for cancer treatment (52–55). The delicate balance of mitochondrial fission and fusion is essential for maintaining functional mitochondrial morphology. Accordingly, excessive fission and fusion lead to mitochondrial dysfunction and cell death. Toxic concentrations of PAI explicitly caused excessive mitochondrial fragmentation, swelling, and clustering in MM and OS cells, whereas only modest mitochondrial fission occurred in fibroblasts (**Figure 7**). Low concentrations of PAI primarily caused mitochondrial fragmentation with minimal swelling and clustering. We found that Sal showed similar effects. Interestingly, substantial mitochondrial swelling and clustering occurred when PAI and Sal were administered together (**Figure 7**). Mitochondrial swelling is a hallmark of the opening of multiple mitochondrial permeability transition pores, and mitochondrial clustering represents an irreversible denature of the mitochondria, which may compromise the mitochondrial network homeostasis. Therefore, swelling and clustering of fragmented mitochondria are critical in cell death, and that exacerbation of these events may play a role in the cooperative actions between PAI and Sal (**Figure 8**).

Both direct NTAPP irradiation and PAL treatment have strengths and weaknesses. Direct plasma treatment is easy to control and chemically defines the active species. However, it is limited to cancers at the body surface because of its short outreach. PAL treatment is a rather indirect and more complicated approach compared to direct plasma irradiation. NTAPP irradiation to liquids results in various active chemical species, which are challenging to define. Nevertheless, PALs may be a promising approach for cancer treatment because they can be readily applied to all types of cancers, including leukemia and lymphomas, by endoscope and infusion. Although PAI induced cell death in MM and OS cells *in vitro* and significantly reduced OS tumor growth *in vivo*, the tumor could not be eliminated, demonstrating a limitation to PAI monotherapy. In clinical practice, it is difficult to treat cancer patients by mono chemotherapy. Considering this preservation of the residual tumor’s aggressiveness, the combination of PAI therapy with a chemotherapeutic drug may be a viable option as a new treatment regime, mainly if the combined drug itself is effective and safe. In the present study, we found that Sal is a candidate that meets these demands. As this compound can selectively kill cancer stem cells and multidrug-resistant cancer cells (33–35), combination use of PAI and Sal may be useful for killing cancer stem cells and multidrug-resistant cancers that are tolerant to multidisciplinary treatment and cause tumor recurrence. Recent studies revealed that Sal causes significant weight loss in mice and toxicity to the male reproductive system and neuronal cells *in vivo* (26). Strikingly, combinatorial administration of Sal and other drugs such as gemcitabine, paclitaxel, and cisplatin ameliorate Sal toxicity and show synergistic effects (26). Therefore, combination therapy with another drug rather than monotherapy is more promising and practical for Sal’s clinical use. In this regard, it is notable that PAI and Sal mutually act as adjuvants to kill OS cells (**Figure 4**). Thus, PAI and Sal’s combined use can significantly reduce effective doses’ windows, thereby preventing adverse effects.

Understanding the components of PAI remains challenging. Various types of analysis methods such as electron spin resonance and high-pressure liquid chromatography may help address this issue. Notably, we observed that PAI was much more effective than H_2_O_2_ (100 μM), which was 2-fold higher than that contained in PAI (50%) (**Figure 1**). These observations suggest that another oxidant participates in the effect of PAI. Considering the chemical composition of infusion fluid, PAI may be similar to plasma-activated PBS, which mainly contains H_2_O_2_, $N{\mathrm{O2}}_{-}$, and $N{\mathrm{O3}}_{-}(4)$. H_2_O_2_ and $N{\mathrm{O2}}_{-}$ can synergistically induce cell death in human colorectal and MM cells. Therefore, nitrogen oxides may play a role in oxidative stress and cell death caused by PAI. Further studies are needed to evaluate these possibilities.

In conclusion, PAI and Sal’s combination administration may be a safe and effective approach for treating apoptosis-resistant cancers such as MM and OS.

## Data Availability Statement

The raw data supporting the conclusions of this article will be made available by the authors, without undue reservation.

## Ethics Statement

The animal study was reviewed and approved by Guidelines for Proper Conduct of Animal Experiments, Science Council of Japan. Protocols were approved by the Animal Care and Use Committee (No.17–11), University of Yamanashi.

## Author Contributions

TA and YS-K designed the experiments, performed the experiments, analyzed data, supervised the completion of this work, wrote the original draft, reviewed and edited the manuscript. MaS-K, MiS-K, JI, and HH performed the experiments and analyzed data. MaS-K, TO, and YY wrote the original draft. TA, TO, YY, HH, and YS-K were responsible for funding acquisition. All authors contributed to the article and approved the submitted version.

## Funding

This research was funded by JSPS KAKENHI; grant numbers 15K09792, 17K10988, 18K08279, and 18K09121.

## Conflict of Interest

The authors declare that the research was conducted without any commercial or financial relationships that could be construed as a potential conflict of interest.

## Supplementary Material

Supplementary Figure 1
**(A)** The rate of cell necrosis (Annexin V-negative and 7AAD-positive) of **Figure 2C**. **(B)** The rate of cell apoptosis (Annexin V-positive and 7AAD-negative) of **Figure 2C**. Data are the mean ± SD (n = 3). **P <* 0.05; ***P <* 0.01 vs. control treated with vehicle. Examples of uncropped western blots **(C)** and quantification **(D)** for cleaved caspase 3, cleaved caspase 8, and GAPDH from **Figure 2F**.

Supplementary Figure 2 Examples of uncropped western blots **(A)** and quantification **(B)** for pRIP-1, pRIP-3, and RIP from **Figure 2G**.

Supplementary Figure 3
**(A)** Examples of uncropped western blots for pP70-S6, P70-S6, pRictor, Rictor, pRaptor, Raptor, pP62, P62, LC3 I/II, and GAPDH from **Figure 3B**.

Supplementary Figure 4
**(A)** Quantification of pP70-S6, P70-S6, pRictor, Rictor, pRaptor, Raptor, pP62, P62, LC3 I/II, and GAPDH from **Figure 3B**.

Supplementary Figure 5 Examples of uncropped western blots **(A)** and quantification **(B)** for pP62, P62 LC3-I/II, and GAPDH from **Figure 4A**.

Supplementary Figure 6
**(A)** Cells positive for EthD-1 of **Figure 4H** were counted and quantified.

Supplementary Figure 7
**(A)** Schematic flowchart diagram showing the experimental design for PAI and Sal treatment of allograft transplants of osteosarcoma cells in mice. LM8 murine osteosarcoma cells (2 × 10^6^ cells/mouse) in 0.1 ml DMEM were injected subcutaneously into the back of the mice on day 0. Three times per week after day 7, PAI and Sal alone or in combination were administered intravenously to 6 mice per group. The mice were weighed, and the size of the primary tumors was measured every week. On day 35, all mice were sacrificed. **(B)** Representative photos of tumor-bearing mice at 4 weeks after inoculation of LM8 cells.
