# Supplementary figures and images for: Combined Anticancer Effect of Plasma-Activated Infusion and Salinomycin by Targeting Autophagy and Mitochondrial Morphology
Source: Front Oncol. 2021 Jun 4;11:593127. doi: 10.3389/fonc.2021.593127 (PMC8212785; doi:10.3389/fonc.2021.593127)

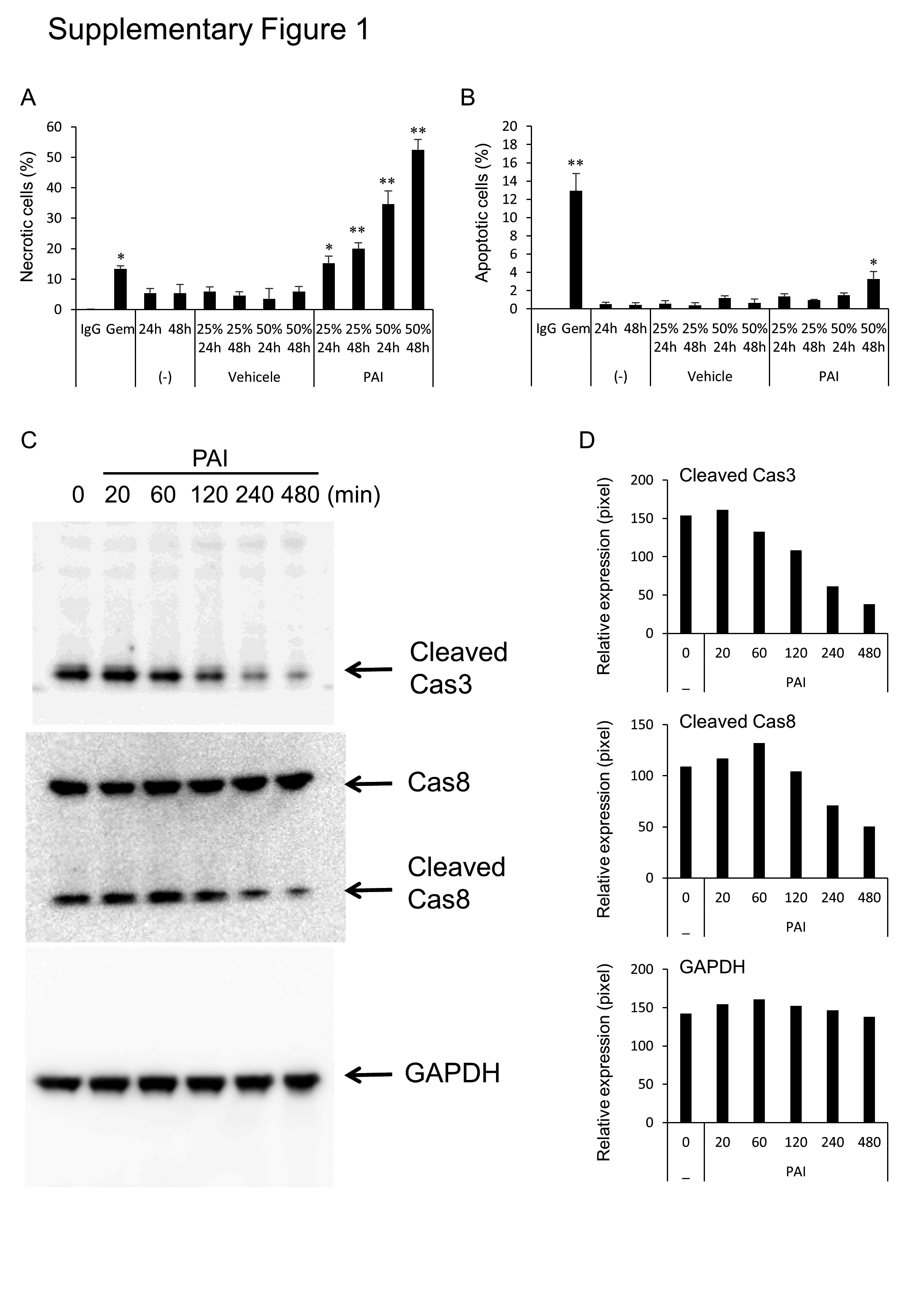

Supplement: Supplementary Figure 1 — (A) The rate of cell necrosis (Annexin V-negative and 7AAD-positive) of Figure 2C . (B) The rate of cell apoptosis (Annexin V-positive and 7AAD-negative) of Figure 2C . Data are the mean ± SD (n = 3). *P < 0.05; **P < 0.01 vs. control treated with vehicle. Examples of uncropped western blots (C) and quantification (D) for cleaved caspase 3, cleaved caspase 8, and GAPDH from Figure 2F . [file Image_1.tif]

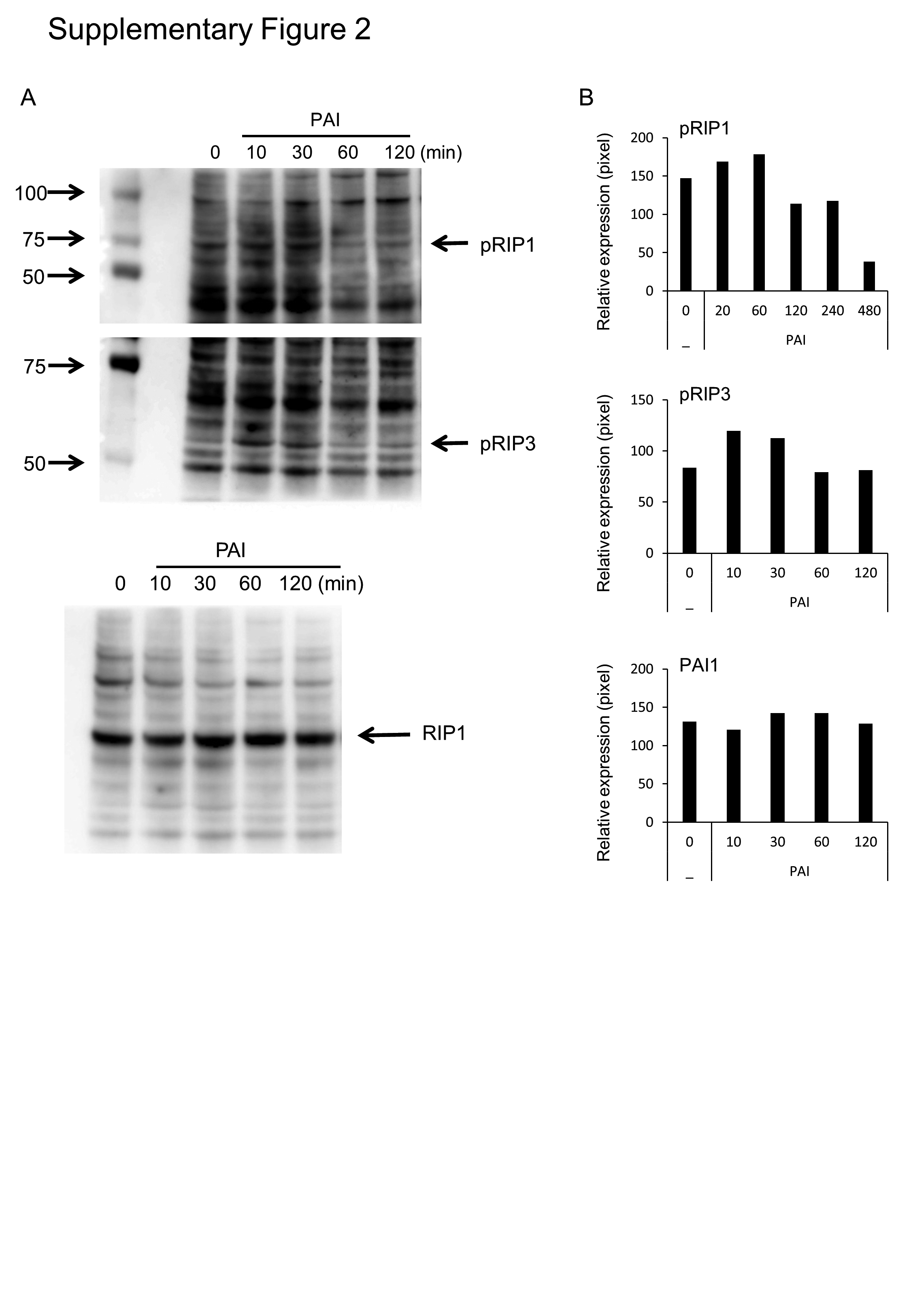

Supplement: Supplementary Figure 2 — Examples of uncropped western blots (A) and quantification (B) for pRIP-1, pRIP-3, and RIP from Figure 2G . [file Image_2.tif]

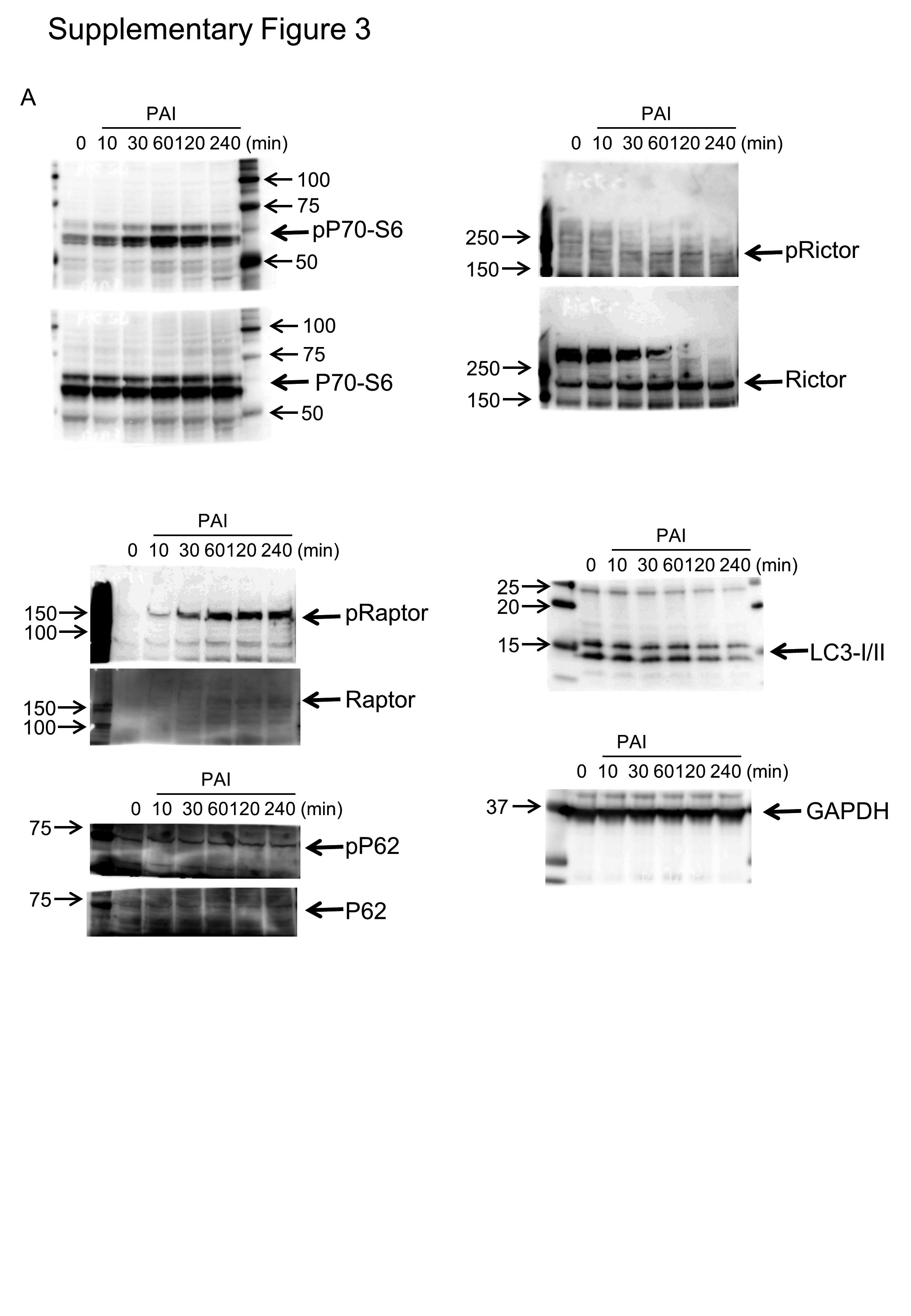

Supplement: Supplementary Figure 3 — (A) Examples of uncropped western blots for pP70-S6, P70-S6, pRictor, Rictor, pRaptor, Raptor, pP62, P62, LC3 I/II, and GAPDH from Figure 3B . [file Image_3.tif]

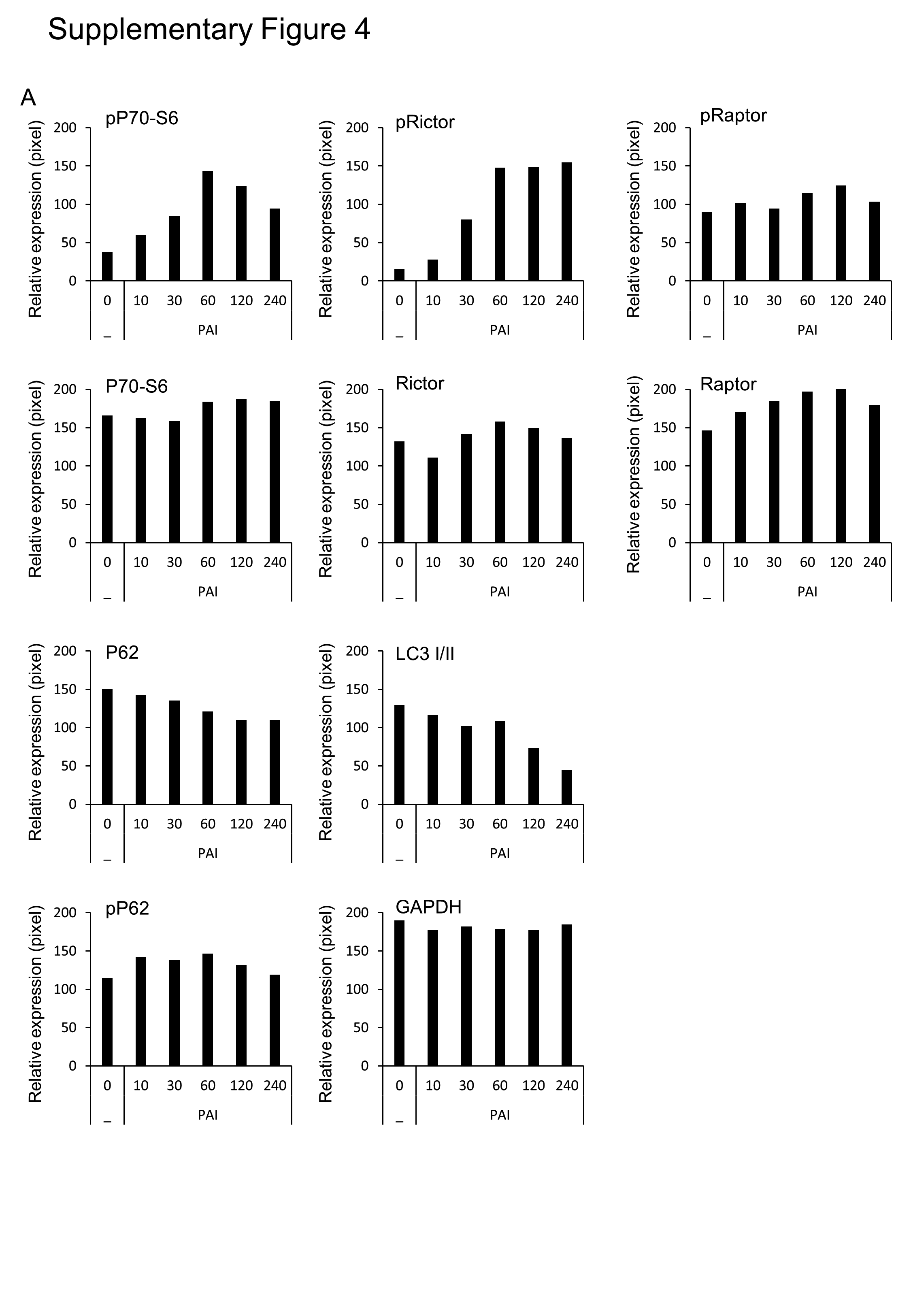

Supplement: Supplementary Figure 4 — (A) Quantification of pP70-S6, P70-S6, pRictor, Rictor, pRaptor, Raptor, pP62, P62, LC3 I/II, and GAPDH from Figure 3B . [file Image_4.tif]

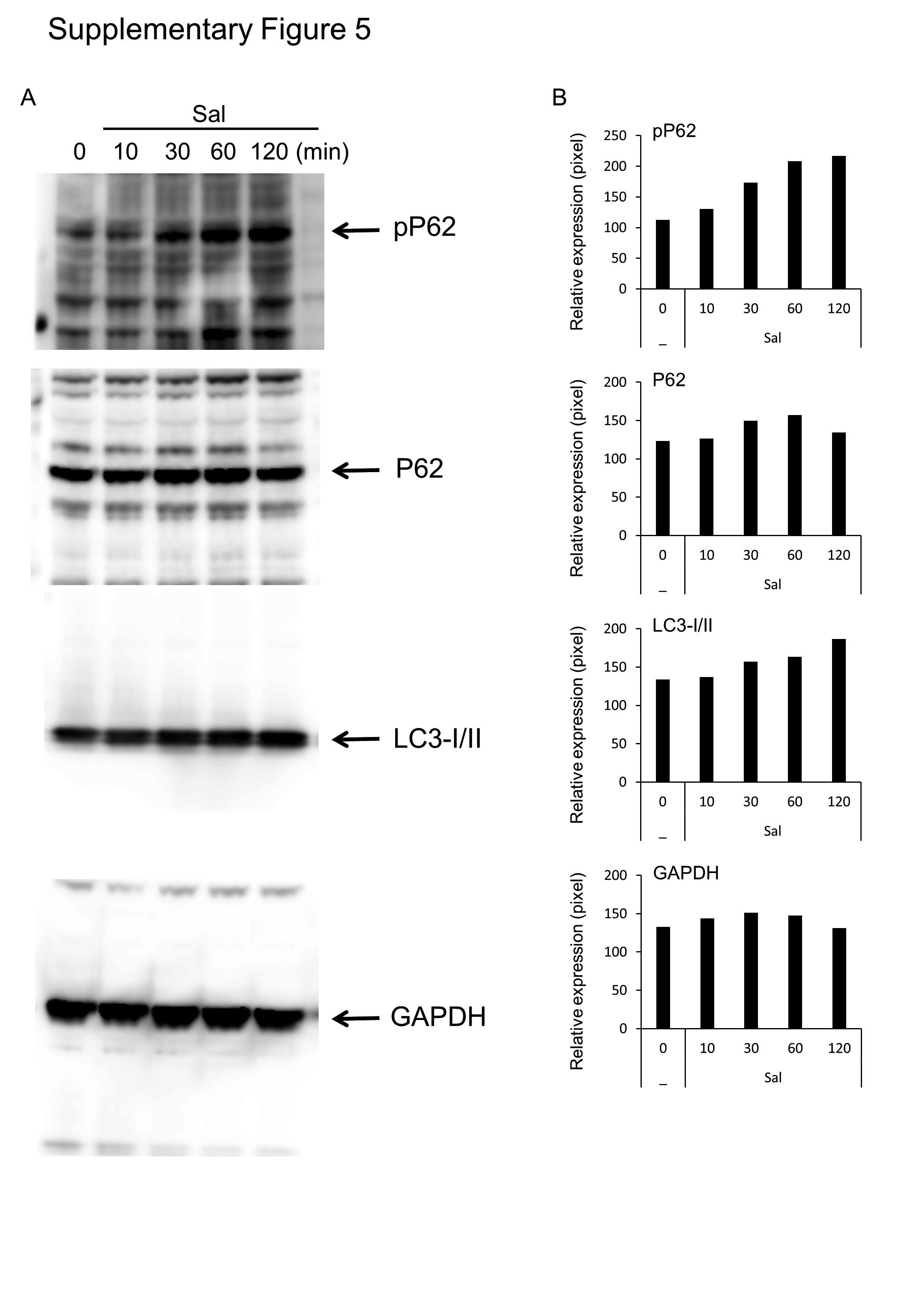

Supplement: Supplementary Figure 5 — Examples of uncropped western blots (A) and quantification (B) for pP62, P62 LC3-I/II, and GAPDH from Figure 4A . [file Image_5.tif]

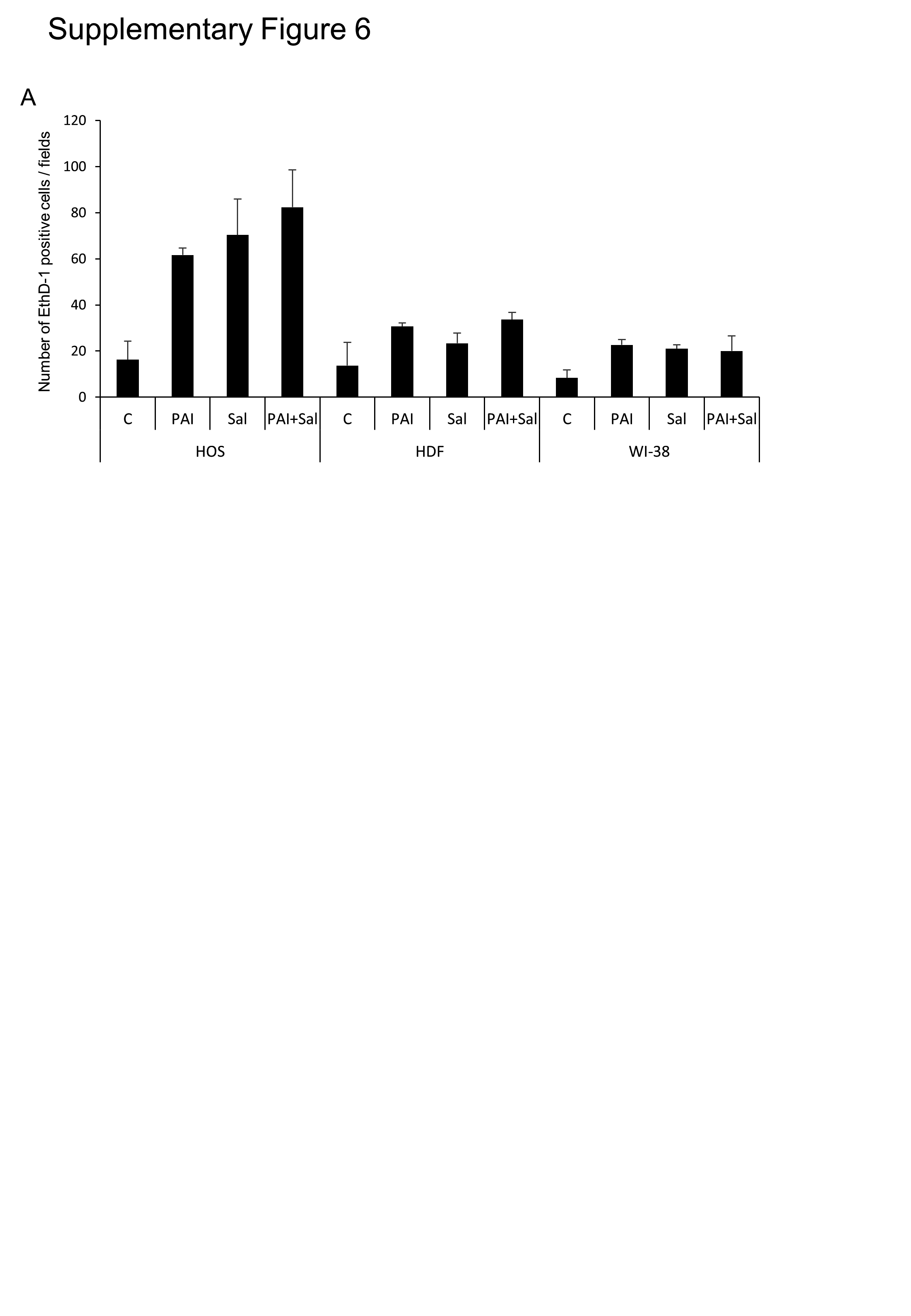

Supplement: Supplementary Figure 6 — (A) Cells positive for EthD-1 of Figure 4H were counted and quantified. [file Image_6.tif]

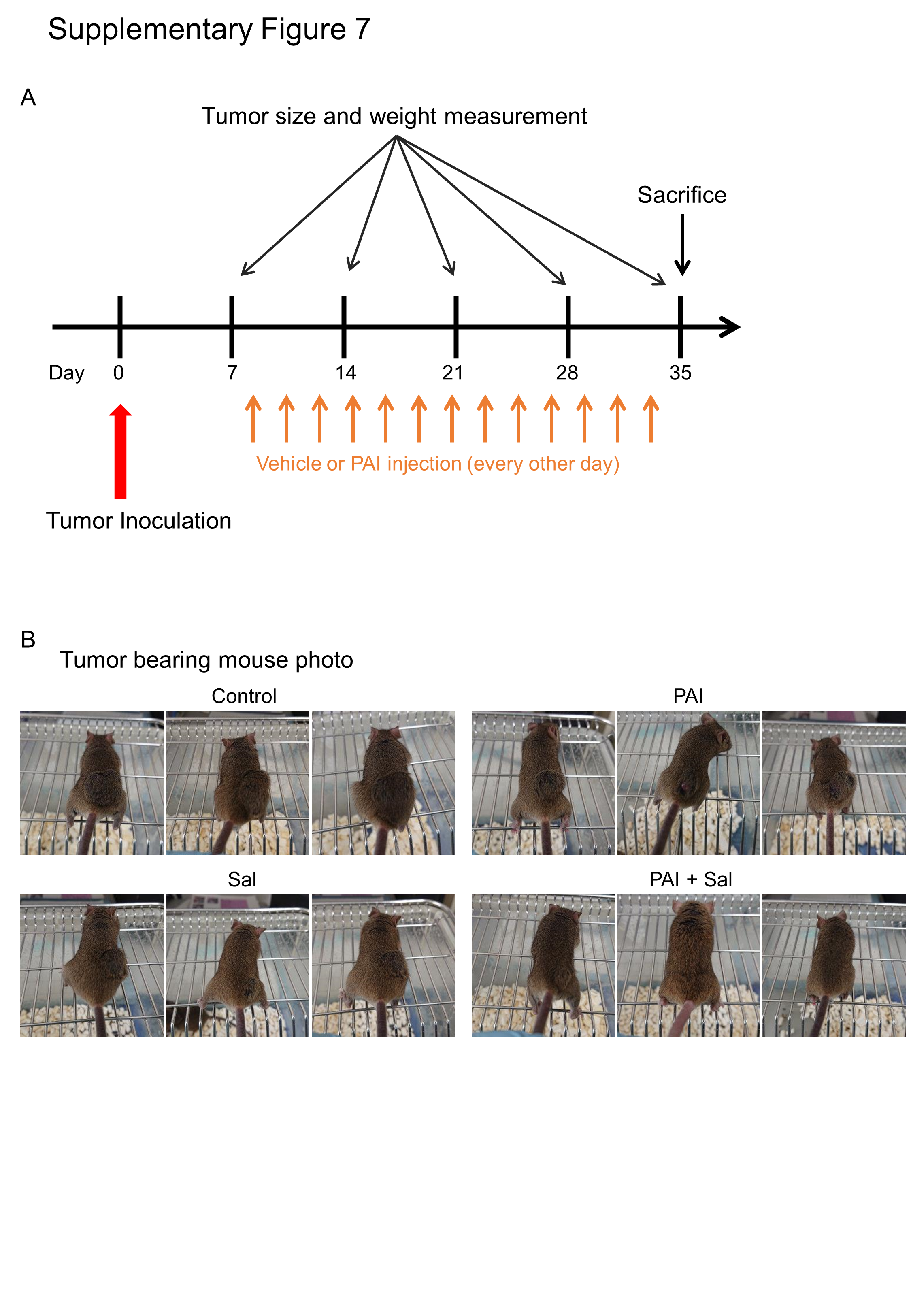

Supplement: Supplementary Figure 7 — (A) Schematic flowchart diagram showing the experimental design for PAI and Sal treatment of allograft transplants of osteosarcoma cells in mice. LM8 murine osteosarcoma cells (2 × 10^6^ cells/mouse) in 0.1 ml DMEM were injected subcutaneously into the back of the mice on day 0. Three times per week after day 7, PAI and Sal alone or in combination were administered intravenously to 6 mice per group. The mice were weighed, and the size of the primary tumors was measured every week. On day 35, all mice were sacrificed. (B) Representative photos of tumor-bearing mice at 4 weeks after inoculation of LM8 cells. [file Image_7.tif]
